# Antidepressants and lethal violence in the Netherlands 1994–2008

**DOI:** 10.1007/s00213-012-2668-2

**Published:** 2012-03-07

**Authors:** Paul F. Bouvy, Marieke Liem

**Affiliations:** 1 Department of Psychiatry, Erasmus Medical Centre, Postbus 2040, 3000 CA Rotterdam, The Netherlands; 2 Institute of Criminal Law & Criminology, Faculty of Law, Leiden University, Postbus 9520, 2300 RA Leiden, The Netherlands

**Keywords:** Epidemiology, Antidepressants, SSRI, Suicide, Homicide, Lethal violence

## Abstract

**Rationale:**

There is an ongoing discussion on the relation between risk of violent behaviour and the use of antidepressants. The claim that the use of antidepressants can cause violent behaviour would gain credibility if a positive association between the two could be established.

**Objective:**

The objective of this study is to evaluate the relationship between homicide, suicide and homicide–suicide rates and the rates of antidepressant use by gender and age group.

**Method:**

Nationwide data from the Netherlands on antidepressant prescriptions (ADs, SSRI and venlafaxine) and lethal violence were analysed over the 15-year period from 1994 to 2008.

**Results:**

The findings indicated a significant negative association between lethal violence (homicide and suicide) and prescription of antidepressants in the Netherlands, indicating that in a period in which the exposure of the Dutch population to antidepressants increased, rates of lethal violence decreased.

**Conclusions:**

These data lend no support for an important role of antidepressant use in lethal violence.

## Introduction

In recent years several publications have suggested a link between the use of antidepressants (ADs)—more specifically SSRIs—and lethal violence including homicide, suicide and homicide–suicide. In case reports and in the lay media, it is frequently suggested that antidepressants can cause serious adverse events, including suicide and violent crime. If this is true, it is important to wage the risk of these possible adverse events against the possible benefits of the treatment with antidepressants. If, however, antidepressant treatment is wrongfully associated with such serious adverse events, these suggestions might lead to a situation in which patients with an indication for treatment with an antidepressant abstain from treatment because of fear for the possibility of these adverse events.

### Suicide

Previous studies on the relation between suicide rates and the prescription of antidepressant data in the USA and in the Nordic countries have shown a decline in suicide rate over a period in which antidepressant sales rose markedly (Grunebaum et al. 2004; Reseland et al. 2006). However, there is an ongoing discussion on the relation between suicide risk and the use of antidepressants in children and adolescents. Although in clinical trials with antidepressants in these groups no or very few completed suicides are reported, there appears to be an increased risk for suicidal ideation and suicidal behaviour (Hammad et al. 2006; Barbui et al. 2009; Stone et al. 2009). This increased risk seems to be present only in participants under 25. In adults aged 25–64, there is a neutral effect and there seems to be a reduced risk of suicidality and suicidal behaviour in those aged over 64 (Stone et al. 2009).

### Homicide and homicide–suicide

Quantitative data on the relationship between violent crime and antidepressants are scarce. One report on homicide–suicides in New York over the years 1990–1998 found that only 3 out of 127 (2.4%) homicide–suicide perpetrators who were tested post-mortem for the presence of an antidepressant tested positive. These data “lend no support to the position that the use of SSRIs is associated with violence or suicide” (Tardiff et al. 2002). Another study on data collected in four US states in the years 2001–2002 showed 15% positive tests in homicide–suicide perpetrators and 19% positive tests on antidepressants in suicide decedents. The authors conclude that these results indicate that there is no link between pharmacotherapy and violent behaviour (Barber et al. 2008).

Other quantitative studies, however, do find significant links between pharmacotherapy and criminal behaviour. For example, crime trends and prescription rates of psycho-pharmaceuticals in the USA over the years 1997–2004 were found to be associated: a rise in the use of antidepressants and stimulants was associated with lower crime rates (Marcotte and Markowitz 2009). A recent study reported on the disproportionally high number of reported violence in the FDA Adverse Event Reporting System for antidepressants with serotonergic effects (Moore et al. 2011).

In other qualitative publications, cases are presented in which a person is using an antidepressant and commits a violent crime. Defendants from the USA, UK, Australia and the Netherlands suggested that antidepressants were to blame for crimes committed (Healy et al. 2006; Mason 2002; Merckelbach et al. 2009) The claim in court that the crime would not have been committed if the antidepressant had not been used is called a counterfactual (Höfler 2005). In other words if the exposure to the antidepressant had not taken place, the incident would not have happened. Here, the exposure to the antidepressant is considered to be a causal factor for the crime to take place. From an epidemiological point of view, “[…] the decisive question is whether the frequency of the undesirable event B will be influenced by a change in the environmental factor A” (Hill 1965). The claim that the use of antidepressants can cause violent behaviour such as homicide would gain credibility if a positive association between the two could be established.

To answer this question, we undertook an epidemiological study in the Netherlands on the use of antidepressants and the rates of homicide, suicide and homicide–suicide in the period 1994–2008. We conducted separate analyses by gender and age group because of the well-known differences between men and women with respect to these forms of violent behaviour and the fact that earlier research suggests that there may be differences between age groups (Stone et al. 2009).

## Methodology

### Data

Various data sources have been used in this study. Data on antidepressant prescriptions come from the GIP, an information system of the Health Care Insurance Board, and is available for the period 1994–2008. It contains information on prescription drugs prescribed by general practitioners and specialists, dispensed by pharmacists, general practitioners and other outlets and are reimbursed under the Health Care Insurance Act. The GIP databases contain data from a representative sample of more than 12 million people (three fourths of the Dutch population). The sample has been obtained from 18 health insurance organisations and has been extrapolated to the size of the entire Dutch population.

Detailed GIP data per type of antidepressant [total antidepressants (AD), SSRI and venlafaxine (Ve)] per age category and gender were, however, only available for the period 2002–2008. In order to calculate the total number of users per type of antidepressant group by gender and age group for the period 1996–2001, we measured the average fraction of users per antidepressant by gender and age group for the period 2002–2008. In this time period (2002–2008), the fraction of users by gender and age remained stable (AD: males 15–30—0.03, males 30–60—0.23, males 60+—0.09; females 15–30—0.06, females 30–60—0.39, females 60+—0.21; SSRI: males 15–30—0.03, males 30–60—0.23, males 60+—0.08; females 15–30—0.07, females 30–60—0.42, females 60+—0.18; Ve: males 15–30—0.03, males 30–60—0.27, males 60+—0.64; females 15–30—0.07, females 30–60—0.42, females 60+—0.14). Subsequently, we applied the fraction of users per antidepressant by gender and group to the period 1996–2001 to calculate the total number of users by gender and age group, to allow for the calculation of user rates per 100,000.

Homicide data were collected from the *Dutch Homicide Monitor*, an ongoing data collection effort that includes the characteristics of incidents, victims and perpetrators of all homicide cases in the Netherlands from 1992 onwards (Nieuwbeerta 2007). Homicide was defined as a lethal offence which has been categorized as either murder (art. 289 and 291 Dutch Code of Criminal Law) or manslaughter (art. 287, 288 and 290 Dutch Code of Criminal Law). The information in this data set is based on various sources, which partially overlap and complement each other and include the following: homicide-related articles generated by the Netherlands National News Agency (ANP), annual summaries of homicides from Elsevier (a weekly magazine), files from the National Bureau of Investigation (NRI), the Public Prosecution Office, the Judicial Information Service, the Ministry of Justice and the Criminal Justice Knowledge Centre (WODC).

Data on homicide—suicide events were also obtained from the Dutch Homicide Monitor and supplemented with additional information stemming from newspaper articles. A homicide—suicide involved a homicide followed by the suicide of the perpetrator within 1 week of the preceding homicide.

Suicide data were retrieved from the dataset *Causes of Death Statistics* from the Dutch Central Bureau of Statistics, Statistics Netherlands (CBS). For the overall population, suicide data were available for the period 1994–2008. For subpopulations by gender and age, data were available from 1996 onwards. There have been no changes in the way suicides were reported in the Netherlands since 1996.

Cases were classified as suicides based on the cause of death given in the certificates from the doctor or forensic pathologist (ICD-10 codes X60-X84). Such officially reported mortality data are considered reasonably sound (Móscicki 1997). Undetermined deaths are not included in the CBS dataset. Not including undetermined deaths leads to underestimation and including leads to overestimation of suicide rates. For the purpose of this study, changes over time are important. The above-mentioned data sets are described in more detail elsewhere (Liem 2010).

### Analysis

Multiple linear regression was used to examine the association between the dependent variables homicide, suicide and homicide–suicide rates and the independent variables of antidepressant use. Antidepressant use was split into three variables: The total use of antidepressants (AD), the total use of SSRIs (SSRI) and the total use of SSRIs including venlafaxine (SSRI+Ve). The reason for combining the use of SSRIs and venlafaxine is research suggesting that venlafaxine acts as an SSRI at 75 mg a day and as a dual 5-HT and NE reuptake inhibitor at higher doses (225 and 375 mg a day, Debonnel et al. 2007).

Total user rates and mortality rates were calculated based on population figures for each year. Separate sets of regression analyses were conducted for each independent variable (homicide rate, suicide rate and homicide–suicide rate). In addition, we conducted analyses on six subgroups by age and gender. Separate sets of regression analyses were conducted per subgroup to determine the relation between antidepressant use and lethal violence. Homicide rates for males above 60 and females in all age categories are extremely low and therefore not reported. The same accounts for the homicide–suicide rate by age and gender subcategories. Given its rare incidence, especially when disaggregated by age and gender category, these subanalyses are not reported (Liem et al. 2009). Statistical analyses were performed with SPSS version 17.0.

## Results

**Fig. 1:**
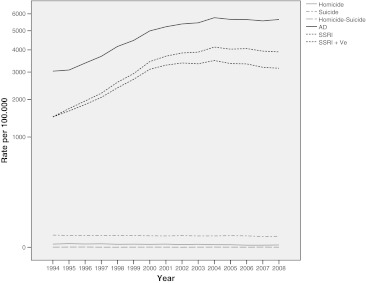
Rates of lethal violence per 100,000 and rates of antidepressant use in the Netherlands, 1994–2008

Our study encompassed nationwide data over a 15-year period. In this period, the homicide rate per 100,000 remained fairly constant, with a moderate decline in recent years, leading to an average of 160 homicide victims per year. The suicide rate decreased steadily from 10.26 (approximately 1,500 victims) in 1994 to 8.77 (approximately 1,400 victims) per 100,000 in 2008. The homicide–suicide rate remained relatively stable, averaging at 0.05 per 100,000 (varying from 4 to 14 victims per year, see Fig. 1). In the same period, the rate of total antidepressant use increased considerably, from 3,038.81 in 1994 to 5,650.58 per 100,000 in 2008. A similar increase was observed for the rate of SSRI use alone, which more than doubled from 1,482.25 in 1994 to 3,161.15 per 100,000 in 2008 as well as the rate of SSRI use including venlafaxine, with an increase of 1,486.81 in 1994 to 3,904.20 per 100,000 in 2008. Over the same period, the number of defined daily doses used rose from 59.6 million in 1994 to 245.3 million in 2008: a fourfold increase. This indicates that not only more people used antidepressants, but also that the mean duration or intensity of the treatment grew (Table 1 and Fig. 1).

**Table 1:** Homicide, suicide and homicide–suicide rates per 100,000 and rates of antidepressant use in the Netherlands, 1996–2008 (95% CI)

Homicide	1.28 (1.14–1.43)
Suicide	9.48 (9.20–9.76)
Homicide–suicide	0.05 (0.04–0.06)
AD	4,745.48 (4,183.68–5,307.28)
SSRI	2,791.73 (2,397.64–3,185.83)
SSRI + Ve	3,149.78 (2,663.89–3,717.67)

### Associations with rates of lethal violence

Linear regression analyses revealed that the total rate of antidepressant use was negatively associated with both homicide rates and suicide rates, but not with homicide–suicide rates alone (see Table 2). Similar results were found between the rates of SSRI use alone and homicide rates as well as suicide rates. Rates of SSRI use and homicide–suicide were not associated.

**Table 2:** Multiple regression analyses for homicide, suicide and homicide–suicide rates per 100,000 and rates of antidepressant use in the Netherlands, 1996–2008 (95% CI)

		Regression coefficient	*P*	95% CI	*R* ^2^
**Total**					
*Homicide*	AD	0.808	<0.001	1.83–2.71	0.653
	SSRI	0.721	0.002	1.59–2.46	0.520
	SSRI + Ve	0.785	0.001	1.63–2.31	0.617
*Suicide*	AD	0.733	0.002	10.23–12.21	0.537
	SSRI	0.669	0.006	9.90–11.72	0.448
	SSRI + Ve	0.725	0.002	9.98–11.44	0.526
*Homicide–suicide*	AD	0.288	0.298	0.02–0.13	0.083
	SSRI	0.290	0.294	0.03–0.12	0.084
	SSRI + Ve	0.271	0.328	0.03–0.11	0.074
**Male 15–30**					
*Homicide*	AD	0.587	0.021	8.70–15.47	0.344
	SSRI	0.586	0.022	8.49–13.63	0.344
	SSRI + Ve	0.645	0.009	8.81–13.18	0.416
*Suicide*	AD	0.839	<0.001	13.51–19.08	0.704
	SSRI	0.801	0.001	12.09–16.57	0.642
	SSRI + Ve	0.831	<0.001	12.10–15.80	0.690
**Male 30–60**					
*Homicide*	AD	0.822	<0.001	4.60–6.59	0.676
	SSRI	0.763	0.001	4.12–5.94	0.582
	SSRI + Ve	0.833	<0.001	4.18–5.49	0.695
*Suicide*	AD	0.381	0.199	11.58–18.92	0.145
	SSRI	0.404	0.152	12.55–18.46	0.163
	SSRI + Ve	0.354	0.235	13.50–18.64	0.126
**Male 60+**					
*Suicide*	AD	0.813	0.001	24.29–35.48	0.660
	SSRI	0.707	0.005	21.45–32.89	0.500
	SSRI + Ve	0.794	0.001	22.27–30.88	0.630
**Female 15–30**					
*Suicide*	AD	0.650	0.016	4.34–8.72	0.422
	SSRI	0.595	0.025	4.01–7.74	0.354
	SSRI + Ve	0.642	0.018	4.14–7.49	0.412
**Female 30–60**					
*Suicide*	AD	0.503	0.080	7.88–13.60	0.253
	SSRI	0.413	0.142	7.53–12.43	0.170
	SSRI + Ve	0.498	0.083	7.95–12.03	0.248
**Female 60+**					
*Suicide*	AD	0.867	>0.001	13.21–18.97	0.751
	SSRI	0.815	>0.001	11.94–17.65	0.664
	SSRI + Ve	0.841	>0.001	11.56–16.06	0.708

Rates of the use of SSRIs including venlafaxine were also found to be negatively associated with both homicide rates and suicide rates. No significant independent association was found between rates of SSRIs including venlafaxine and homicide–suicide.

### Associations with rates of lethal violence by gender and age

**Fig. 2:**
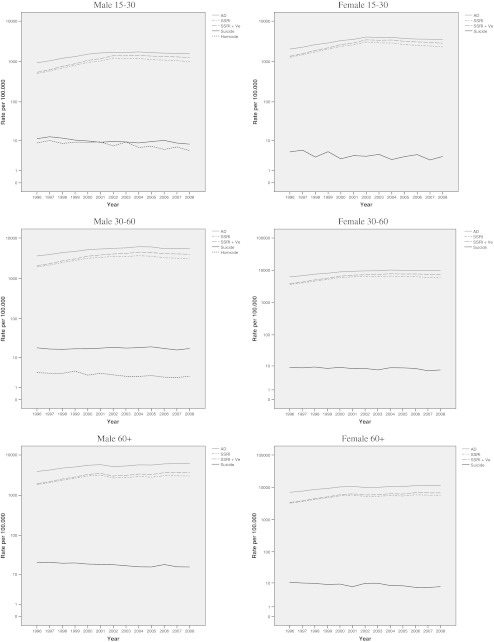
Rates of lethal violence per 100,000 and rates of antidepressant use in the Netherlands, 1996–2008 by gender and age categories

Figure 2 reflects the relationship between total antidepressant use (AD) and rates of lethal violence by gender and age category. The results from the overall regression analyses were replicated (see Table 2): In virtually all categories, there was a significant negative relationship reported between antidepressant use and suicide, with the exception for males aged 30–60, where no relationship in either direction was found. Similarly, there was either a significant negative relationship between the rate of antidepressant use and homicide rate (males aged 30–60) or no significant relationship in either direction (males aged 15–30).

The relationship between SSRI alone and rates of lethal violence per subcategory displayed similar tendencies: There was either a negative relationship between suicide and SSRI alone, or no relationship in either direction (males and females aged 30–60). SSRI use was negatively associated with the homicide rate (males aged 15–30 and males aged 30–60). These results were replicated for SSRI including venlafaxine (SSRI+Ve).

## Discussion

### Suicide

In the period under study, the suicide rate in the Netherlands decreased from 10.26 in 1994 to 8.77 per 100,000 in 2008. This decrease seems to be a continuation of a long-term decline since the 1980s, in which the suicide rate was approximately 15 per 100,000 (van Hemert and de Kruif 2009).

The increase in the use of antidepressants is associated with a decrease in suicide rate. The association is strong; the changes in the use of antidepressants can account for 54% of the variation in suicide rate. These findings are in line with previous research. When examining the younger subgroups that might be especially at risk, the negative association between prescription of AD and SSRI ± Ve on the one hand and suicide rates on the other is confirmed. The increase in suicide risk in children and adolescents found in previous research is largely based on an increase in suicidal ideation and nonfatal suicidal behaviour in studies comparing efficacy of AD versus placebo (Hammad et al. 2006; Stone et al. 2009). The risk factors for nonfatal suicidal behaviour (e.g. younger and female) and completed suicide (e.g. older and male) are not the same: An increase in suicidal ideation and nonfatal suicidal behaviour does not necessarily reflect an increase in completed suicide (Baldessarini et al. 2006).

### Homicide and homicide–suicide

To our knowledge we are the first to report on the relation between lethal violence directed towards others and the use of antidepressants over a long period of time (15 years). We found a significant negative association between homicide and prescription of AD and SSRI ± Ve in the Netherlands, indicating that in a period in which the exposure of the Dutch population to antidepressants increased, the homicide rates decreased. As is the case for suicide, the association is strong, changes in the use of antidepressants can account for 65% of the variation in homicide rates. These data lend no support for an important role of antidepressant use in homicide. With regard to the subgroup analysis, the younger male groups are of primary interest. Here, the same negative associations are found.

These findings are in contrast with a recent study based on reported adverse events in the Adverse Event Reporting System of the FDA from 2004 to 2009, which concludes that “acts of violence towards others are a genuine and serious adverse drug event associated with a relatively small group of drugs [including] antidepressants with serotonergic effects” (Moore et al. 2011). In this study, violent thoughts and acts towards others were significantly more reported than would be expected by chance. There are several ways to explain these findings. Publicity about the possibility of violent behaviour as an adverse event of a class of drugs will raise the number of reports. In addition, one has to take the reasons for prescribing antidepressants into account. Despair can be one reason to consider such treatment. At the same time, despair is a risk factor for violent thoughts and behaviour. Further, problems with impulse control may be a reason to consider antidepressant treatment. Recently, the first placebo-controlled trial was published in which efficacy was shown of an SSRI (fluoxetine) in conjunction with cognitive behavioural therapy in a group of perpetrators of domestic violence. Measures of anger and physical aggression were reduced (George et al. 2011). These findings suggest that antidepressant treatment might actually help patients to control their violent thoughts and behaviour. Earlier research has shown that aggressive behaviour both to oneself or outwards to others is associated with serotonergic dysfunction. Enhancement of serotonergic function by prescription of an antidepressant might be an appropriate thing to do and explain of the negative associations found in the present study (Walsh and Dinan 2001).

There appears to be a striking parallel with the use of antidepressants and suicide. However, suicide has a more direct relationship with depression and the use of antidepressants than homicide. Antidepressants are not generally prescribed to homicidal persons. The negative association found between the use of antidepressants and homicide is probably more strongly influenced by other factors.

### Limitations

Detailed information on individuals who committed a form of lethal violence was not available. Future research should attempt to overcome this difficulty by obtaining additional information on autopsy reports from suicide decedents, an approach used in previous studies (Barber et al. 2008). In addition, detailed data per type of antidepressant per age and gender category were only available for the period 2002–2008. By applying average fractions of users per antidepressants, we were able to calculate the total number of users by gender and age group for the period 1996–2001. This approach, however, is based on the assumption that the fraction of users per antidepressants remains fairly stable over time. This assumption might have caused a wrongful estimation of actual use per age and gender category.

## Conclusion

Despite these limitations, this study relies on nationwide data and is the first to report on the relation between different forms of lethal violence and the use of antidepressants over long period of time. The findings for homicide and suicide appear to have much in common. However, violent acts such as homicide, suicide and homicide–suicide are complex forms of human behaviour in which many factors may play a role (Luo et al. 2011). Medication is only one of them. One should be cautious to draw simple conclusions. The associations found point to a beneficial effect of antidepressants on these very serious forms of aggression.
